# The Impact of TRPM8 on Prostate Cancer Transcriptomic Dynamics

**DOI:** 10.3390/cells14070501

**Published:** 2025-03-27

**Authors:** Swapna Asuthkar, Susovon Bayen, Erick B. Saldes, Benny Tom, Jai Velpula, Sarangi Siddharth, Timothy E. Koeltzow, Donald J. Vander Griend

**Affiliations:** 1 Department of Cancer Biology and Pharmacology, University of Illinois College of Medicine Peoria, Peoria, IL 61605, USAesaldes@uic.edu (E.B.S.); btom4@uic.edu (B.T.); siddharth.sarangi@osfhealthcare.org (S.S.); tkoeltzow@fsmail.bradley.edu (T.E.K.); 2 Department of Pediatrics, University of Illinois College of Medicine Peoria, Peoria, IL 61605, USA; 3 Pringle Robotics, Peoria, IL 61614, USA; jaivelpula@gmail.com; 4 OSF Saint Francis Medical Center, Peoria, IL 61637, USA; 5 Department of Psychology, Bradley University, Peoria, IL 61625, USA; 6 Department of Pathology, University of Illinois at Chicago, Chicago, IL 60614, USA; dvanderg@uic.edu

**Keywords:** TRPM8, mRNA expression, androgen, androgen receptor (AR), prostate cancer

## Abstract

Prostate cancer (PC) remains a significant health challenge, with androgen receptor (AR) signaling playing a pivotal role in its progression. This study investigates the expression and functional implications of the transient receptor potential melastatin 8 (TRPM8) channel in PC, focusing on its interaction with AR and its impact on oncogenic pathways. We analyzed mRNA expression levels of TRPM8 and AR in PC tissues, revealing that TRPM8 is upregulated in benign and early-stage tumors but significantly downregulated in metastatic samples. This decline correlates with increased AR expression, suggesting a compensatory mechanism that enhances AR-driven tumorigenesis. RNA sequencing and pathway enrichment analyses demonstrated that TRPM8 knockout (KO) prostates exhibited significant alterations in gene expression, particularly in pathways related to extracellular matrix (ECM) remodeling, cell proliferation, and survival signaling. Notably, genes associated with metastasis, such as MMP2 and FAP, were upregulated in TRPM8 KO samples, indicating a potential role for TRPM8 in inhibiting tumor invasion. Furthermore, Gene Set Enrichment Analysis (GSEA) revealed positive enrichment of androgen response, angiogenesis, and epithelial–mesenchymal transition (EMT) pathways in TRPM8 KO prostates, reinforcing the notion that TRPM8 loss creates a pro-tumorigenic environment. Our findings suggest that TRPM8 functions as a molecular brake on PC progression, and its loss may contribute to the development of aggressive disease phenotypes. This study underscores the importance of TRPM8 as a potential therapeutic target and biomarker in PC, warranting further investigation into its role in cancer biology and treatment response.

## 1. Introduction

Prostate cancer (PC) persists as one of the most commonly occurring malignancies in the male population, with its pathophysiological mechanisms predominately associated with androgen signaling pathways. The androgen receptor (AR) is crucial in the development and progression of PC, functioning as a transcription factor that controls genes essential for cell growth and survival [1]. Over the past few decades, the understanding of AR’s role has evolved significantly, particularly with the advent of androgen deprivation therapy (ADT) and, more recently, androgen receptor signaling inhibitors (ARSIs), such as enzalutamide (ENZ) and abiraterone (ABI), which have become cornerstones in managing advanced PC [2,3]. These therapies aim to either reduce androgen levels and/or block AR signaling, thereby slowing tumor progression [4]. However, the emergence of castration-resistant PC (CRPC) poses a significant challenge, as tumors often adapt to low androgen environments, leading to treatment resistance and disease progression [5].

Recent studies have highlighted the importance of ion channels, particularly transient receptor potential melastatin 8 (TRPM8), in PC biology. TRPM8, a calcium-permeable channel activated by cold temperatures and menthol, is implicated in various cellular processes, including proliferation, apoptosis, and migration. TRPM8 expression in prostate tissues is complex; it is upregulated in early-stage tumors but declines in metastatic disease, suggesting a potential role in tumor suppression during early tumorigenesis [6,7]. This paradoxical behavior raises questions about the functional significance of TRPM8 in the context of androgen signaling and its potential as a therapeutic target. TRPM8 has been shown to interact with AR, influencing its activity and potentially modulating cancer progression [6,8,9].

The interaction between TRPM8 and AR signaling is particularly intriguing. Evidence suggests that TRPM8 may act as a testosterone receptor, mediating some effects of androgens on PC cells [6,7,10]. This relationship is further complicated by the observation that TRPM8 expression is modulated by AR activity, indicating a feedback mechanism that could influence cancer progression. For instance, TRPM8 degradation in PC cells has been linked to increased AR activity, which may facilitate tumor growth and metastasis [6,11]. Moreover, the differential expression of TRPM8 and AR across various stages of PC, particularly in response to ARSIs, underscores the need for a comprehensive analysis of their roles in disease progression. As PC evolves, the balance between TRPM8 and AR expression may dictate the tumor’s response to therapy and its metastatic potential.

In summary, the intricate relationship between TRPM8 and AR signaling in PC presents a promising avenue for research, with potential implications for improving treatment outcomes in patients with advanced disease. Understanding the molecular mechanisms regulating TRPM8 and its interaction with AR could uncover novel therapeutic strategies to re-sensitize tumors to ADT/ARSIs or target the TRPM8-AR axis directly. Moreover, by investigating the expression patterns of TRPM8 and AR and their functional implications on PC progression, this study aims to enhance therapeutic approaches and improve outcomes for patients with advanced PC.

## 2. Materials and Methods

### 2.1. Ethics Statement

All human patient tissue mRNA data were obtained from the University of Illinois Cancer Center, Chicago, Illinois, and processed in accordance with Dr. Vander Griend’s Lab Institutional Review Board-approved protocol (protocol # 2019-0061). For the mice data, The Institutional Animal Care and Use Committee of the University of Illinois College of Medicine at Peoria, Peoria, IL, USA, approved all surgical interventions and post-operative animal care. The consent was written and approved. The accepted protocol number is 1807728, dated 6 October 2021, and renewed on 8 November 2024.

### 2.2. Animals and Housing Conditions

Male C57BL/6J (# 000664) and TRPM8 knockout (KO, # 008198) mice were obtained from The Jackson Laboratories (Bar Harbor, ME, USA) and were directly utilized for RNA-sequencing analysis. The TRPM8 KO mice were originally donated to Jackson Laboratories by Dr. David Julius’ laboratory [12]. These mice undergo regular genotype evaluations in accordance with the Jackson Laboratory genotyping protocol. In our study, we maintained the mice without any breeding. Mice were housed in groups of 2–7 per cage in yellow-tinted plastic cages (43.2 cm length × 20.3 cm width × 20.3 cm height) containing structural enrichment (nesting material and a cardboard tube/hiding place) at the University of Illinois College of Medicine in Peoria. Mice were kept under a 12/12 light/dark cycle (lights on at 06:00 and off at 18:00 h) under consistent temperature and humidity (22.8 ± 2.0 °C, 45–50% humidity) with food and water available ad libitum (LabDiet 5LG4). At 14 weeks old, mice were euthanized by CO_2_ asphyxiation and cervical dislocation.

### 2.3. RNA Sequencing

Total RNA was extracted from TRPM8 knockout (KO) (*n* = 6) and wild-type (WT) (*n* = 6) mouse prostate tissues using TRIzol™ Reagent (Thermo Fisher Scientific, Waltham, MA, USA) following the manufacturer’s protocol. The RNA quality and quantity were assessed using a Bioanalyzer (DS-11FX DeNovix, Wilmington, DE, USA) and NanoDrop ND-1000 (Thermo Fisher Scientific, Waltham, MA, USA), respectively. Samples with OD260/280 ratios between 1.8 and 2.1 were deemed acceptable. Approximately 1–2 µg of total RNA was used for library preparation for KO (*n* = 2) and WT (*n* = 2). Libraries were prepared using the KAPA Stranded RNA-Seq Library Prep Kit (Illumina, San Diego, CA, USA), incorporating dUTP into the second cDNA strand to ensure strand specificity. Libraries were qualified on an Agilent 2100 Bioanalyzer and quantified by qPCR. Sequencing was performed on the Illumina NovaSeq 6000 platform at Arraystar Inc. (Rockville, MD, USA), generating paired-end reads of 150 base pairs.

### 2.4. Data Analysis

Raw sequencing data underwent quality control using the software FastQC (version 0.11.7), and adapters were trimmed with Cutadapt. Reads were aligned to the GRCm38 reference genome using Hisat2. Mapping statistics, including mapping ratios and rRNA content, were analyzed to ensure high-quality data. Transcript abundances were estimated with StringTie, and differential expression analysis was conducted using Ballgown. Differentially expressed genes (DEGs) were identified based on a fold change cutoff of 1.5 and a *p*-value ≤ 0.05.

### 2.5. Pathway Enrichment Analysis

Pathway enrichment analysis was performed to identify biological processes affected by TRPM8 deletion. Gene ontology (GO) enrichment analysis categorized DEGs into biological processes (BPs), molecular functions (MFs), and cellular components (CCs). KEGG pathway analysis identified enriched pathways among DEGs, with significance determined using Fisher’s exact test. Gene Set Enrichment Analysis (GSEA) was employed to assess predefined gene sets for significant differences between KO and WT samples.

### 2.6. Data Visualization Statistical Analysis

Data visualization included hierarchical clustering, Principal Component Analysis (PCA), scatter plots, and volcano plots to illustrate gene expression differences between KO and WT samples. Alternative splicing events were detected using rMATS (version 4.0.1), categorizing events into alternative 3′ splice site (A3SS), alternative 5′ splice site (A5SS), skipped exons (SEs), mutually exclusive exons (MXEs), and retained introns (RIs). Visualization of splicing events was performed using rmats2sashimiplo (Version 2.0.2)t. Novel genes and transcripts were identified using StringTie (Version 1.3.3) and Ballgown (Version 2.10.0), and the coding potential of novel transcripts was assessed using the Coding Potential Assessment Tool (CPAT). Statistical analyses were performed using R (Version 3.5.0), Python (Version 2.7), and Minitab software Version 21.1.0 (Minitab, State College, PA, USA). Statistical differences are presented at ns, *p* > 0.05; *, *p* < 0.05; **, *p* < 0.005; ***, *p* < 0.0001. Data were presented as means ± standard deviation (SD) or standard error of the mean (SEM) where applicable.

## 3. Results

### 3.1. TRPM8 and AR mRNA Expression in Prostate Cancer and Implications for Androgen-Deprivation Therapies

**Figure 1:**
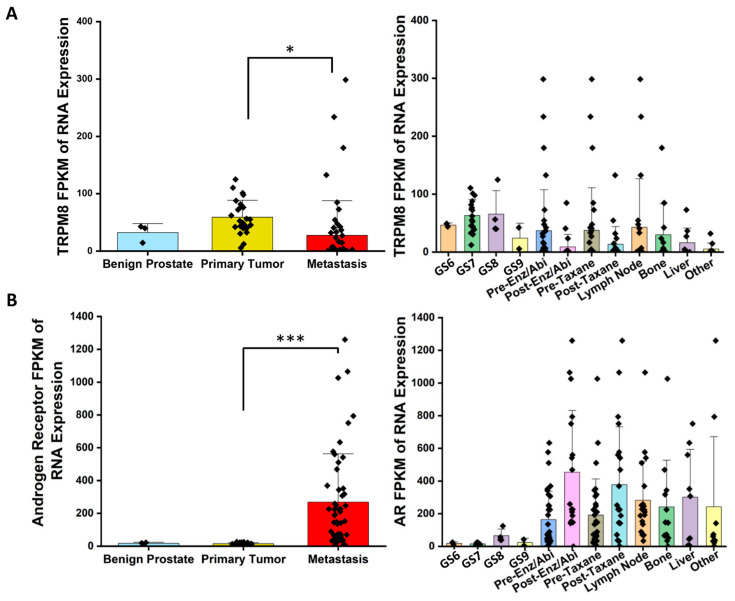
mRNA expression of TRPM8 and AR in PC patients from the UIC Cancer Center. (**A**) TRPM8 mRNA expression: bar graphs display fragments per kilobase of transcript per million mapped reads (FPKM) for TRPM8 in benign, tumor, and metastatic prostate tissues. The right panel provides FPKM values for individual patient samples. (**B**) AR mRNA expression: bar graphs show FPKM values for AR across benign, tumor, and metastatic tissues, with individual sample data on the right (Gleason score = GS, GS6 = low grade, GS7 = intermediate, and GS8–9 = high grade) (ANOVA with post hoc Tukey multiple comparisons of different tissues (*, *p* < 0.05, ***, *p* < 0.0001; *n* = 3–51)).

The analysis of mRNA expression levels for TRPM8 and AR in PC patients from the UIC Cancer Center reveals distinct patterns across benign, tumor, and metastatic stages, providing insights into their roles in cancer progression and androgen signaling regulation. As shown in Figure 1A, TRPM8 expression is significantly elevated in tumor tissues compared to benign samples, suggesting its involvement in early-stage prostate tumorigenesis [6,7,13]. However, TRPM8 expression declines sharply in metastatic samples, indicating a potential reduction in its regulatory function as cancer advances. In contrast, Figure 1B shows that AR expression is markedly higher in both tumor and metastatic tissues relative to benign samples, underscoring AR’s central role in driving PC [14]. This inverse relationship between TRPM8 and AR expression may imply a compensatory mechanism, where the downregulation of TRPM8 is associated with heightened AR activity, particularly in advanced cancer stages. The increase in AR expression as TRPM8 levels drop could highlight a shift toward AR-driven progression, a phenomenon often associated with metastatic and CRPC. The figure also displays TRPM8 and AR expression variations in response to the Gleason score and ARSIs, like ABI and ENZ. Tissues with higher Gleason scores and those treated with ARSIs show reduced TRPM8 expression, possibly due to suppressed androgen signaling (Figure 1A). Conversely, AR expression remains high or increases post-ARSI, suggesting that AR-driven pathways continue to dominate, potentially contributing to CRPC development (Figure 1B).

Our previous studies showing elevated serum testosterone levels in TRPM8 knockout (TRPM8 KO) mice further support these findings, suggesting that the loss of TRPM8 may disrupt androgen homeostasis [15]. Elevated testosterone in TRPM8 KO mice could lead to unchecked AR activity, reinforcing the link between the loss of TRPM8 and enhanced AR signaling in PC progression [13]. These findings from human samples, combined with our previous work demonstrating AR-mediated TRPM8 degradation [7], suggest a potential mechanism for the observed inverse correlation. Specifically, AR activation may lead to TRPM8 protein downregulation, which in turn enhances androgen–AR activity, creating a positive feedback loop that promotes tumor progression. The reduction in TRPM8 in metastatic tissues, alongside elevated AR levels, suggests that the TRPM8–AR–androgen axis is crucial in PC progression. Targeting this axis to restore TRPM8 activity could help counteract AR-driven tumorigenesis, particularly when ADT/ARSI efficacy diminishes, offering insights for personalized therapeutic strategies.

### 3.2. Role of TRPM8 in Prostate Cancer via RNA Sequencing of TRPM8 Knockout and Wild-Type Prostate Tissues

RNA sequencing was performed to investigate the role of TRPM8 in PC and assess the tumorigenic potential of TRPM8 KO compared to wild-type (WT) mouse prostates. High-quality total RNA was extracted from WT and TRPM8 KO prostate tissues, which was confirmed by the presence of sharp and distinct 28S and 18S ribosomal RNA bands in agarose gel electrophoresis (Supplementary Figure S1A). The clear resolution of these bands, with the 28S band approximately twice as intense as the 18S band, indicates intact RNA, ensuring high-quality samples for downstream analysis [16,17]. The sequencing workflow, outlined in Supplementary Figure S1B, included mRNA enrichment, library preparation using Illumina kits, and paired-end sequencing on the Illumina platform at Arraystar Inc. This approach enables comprehensive transcriptome coverage, allowing for an in-depth gene expression analysis in WT and TRPM8 KO prostate tissues. The bioinformatics pipeline incorporated stringent quality control steps, using FastQC for quality assessment and Cutadapt for adapter trimming. Reads were aligned to the GRCm38 reference genome with Hisat2, an alignment tool optimized for spliced reads, essential for accurate prostate transcriptome mapping. Transcript abundances were quantified using StringTie, followed by differential expression analysis with the Ballgown package, identifying differentially expressed genes between WT and TRPM8 KO prostates based on a fold change cutoff of 1.5 and a *p*-value ≤ 0.05 (Supplementary Figure S1C). This analysis revealed significant differences in gene expression profiles between WT and TRPM8 KO samples, highlighting the molecular impact of TRPM8 deletion on prostate tissue. Pathway enrichment analysis identified key pathways related to cell proliferation, survival, and cancer-related signaling, which were differentially regulated in TRPM8 KO prostates. These findings suggest that TRPM8 plays a crucial role in maintaining normal gene expression patterns in the prostate, and its loss may drive changes that contribute to tumor progression.

### 3.3. Differential Gene Expression and Pathway Alterations in TRPM8 Knockout Prostates

**Figure 2:**
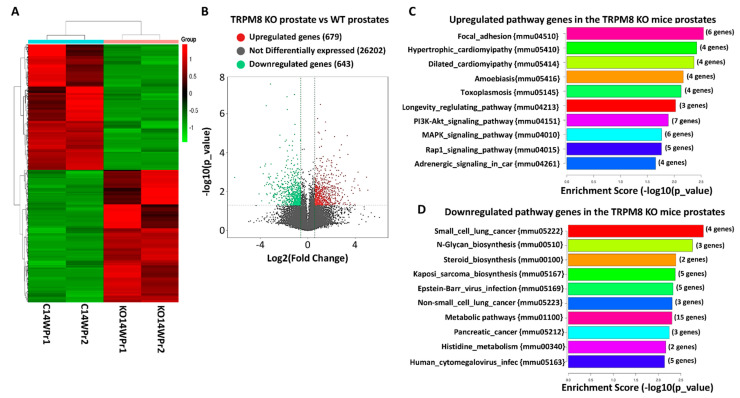
Differential gene expression and pathway enrichment analysis of TRPM8 KO and WT mouse prostates. (**A**) Hierarchical clustering of differentially expressed genes between TRPM8 KO (*n* = 2) and wild-type (WT) (*n* = 2) mouse prostates. Red indicates upregulated genes and green indicates downregulated genes. (**B**) Visualization of differentially expressed genes with log2 fold change on the *x*-axis and −log10 *p*-value on the *y*-axis. Red and green dots represent significantly upregulated and downregulated genes, respectively, with a fold change cutoff of 1.5 and *p*-value ≤ 0.05. (**C**) Displays the top ten enriched pathways of differentially expressed genes (DEGs) in TRPM8 KO compared to WT mouse prostates. The enrichment score is presented as −log10 (*p*-value), highlighting significant pathways, such as focal adhesion and PI3K-Akt signaling. (**D**) Displays the top ten downregulated pathways in TRPM8 KO compared to WT mouse prostates. The enrichment score is presented as −log10 (*p*-value), highlighting significant pathways, such as small cell lung cancer and N-glycan biosynthesis.

RNA sequencing analysis of WT and TRPM8 KO mouse prostates revealed distinct gene expression profiles, highlighting the impact of TRPM8 deletion on gene regulation in prostate tissue. As shown in Figure 2A, the heatmap displays hierarchical clustering of differentially expressed genes, with clear segregation between WT and TRPM8 KO samples. This clustering indicates substantial alterations in gene expression patterns, suggesting that TRPM8 plays a role in maintaining normal gene expression in the prostate. The volcano plot (Figure 2B) illustrates the range of expression changes, identifying genes that meet the differential expression criteria with a log2 fold change greater than 1.5 and a *p*-value ≤ 0.05. This analysis identified 679 upregulated and 643 downregulated genes in TRPM8 KO compared to WT samples, underscoring significant shifts in gene activity associated with TRPM8 loss. Further analysis revealed that differentially expressed genes are enriched in cell proliferation, survival, and cancer progression pathways. The observed changes suggest that TRPM8 deletion impacts key regulatory pathways, potentially contributing to a pro-tumorigenic environment in the prostate.

### 3.4. Pathway Enrichment Analysis of Upregulated Genes in TRPM8 Knockout Prostates

Compared to WT controls, the pathway enrichment analysis of upregulated genes in TRPM8 KO mouse prostates revealed significant alterations in several key signaling pathways. The bar plot (Figure 2C) highlights the top ten enriched pathways, with focal adhesion, hypertrophic cardiomyopathy, PI3K-Akt, and MAPK signaling among those with the highest enrichment scores [18]. These pathways are crucial for maintaining cell–matrix interactions, growth, and survival, suggesting that the loss of TRPM8 significantly impacts processes that promote tumor progression and metastasis. The focal adhesion pathway, enriched with genes, such as *Col4a5*, *Flnc*, *Igf1*, *Lama2*, and *Pdgfra* (detailed in Table 1), plays a central role in cell adhesion and interaction with the ECM [19,20,21]. The upregulation of this pathway in TRPM8 KO prostates suggests enhancing cell adhesion dynamics, which could facilitate cancer cell migration and invasion. Similarly, the PI3K-Akt signaling pathway, critical for cell survival and proliferation, includes upregulated genes, such as *Igf1* and *Pdgfra*. This pathway’s activation indicates an increased capacity for cellular growth and resistance to apoptosis in the absence of TRPM8, potentially contributing to tumor development [22,23,24]. Other upregulated pathways, like MAPK signaling and Rap1 signaling, are also implicated in cell proliferation and survival, further highlighting TRPM8’s potential role in suppressing these cancer-promoting pathways [25,26]. Table 1 further details the enriched pathways, listing the specific genes involved. The upregulation of these genes in pathways associated with cell adhesion, signaling, and growth underscores the impact of TRPM8 deletion on key regulatory mechanisms. These findings indicate that the loss of TRPM8 may contribute to a pro-tumorigenic environment by enhancing pathways that support cellular proliferation, survival, and metastasis.

### 3.5. Pathway Enrichment of Downregulated Genes in TRPM8 Knockout Prostates

The pathway enrichment analysis of downregulated genes in TRPM8 KO mouse prostates, as shown in Figure 2D, reveals significant suppression of pathways crucial for cell cycle regulation, apoptosis, steroid biosynthesis, and metabolism, suggesting that the loss of TRPM8 leads to significant molecular alterations that may facilitate cancer progression. Table 2 lists the genes downregulated in these pathways. Metabolic pathways are among the most significantly affected, with key genes such as *Aldh3b2*, *Amdhd1*, *Acyp1*, *Arg1*, and *Cbs* being downregulated [27,28]. These genes are involved in amino acid metabolism, detoxification, and cellular energy balance, all essential for normal cellular function and growth control. The suppression of these pathways points to metabolic reprogramming commonly observed in cancer cells, where they shift their metabolism to support increased proliferation and survival under nutrient-limited conditions. This disruption in energy and biosynthetic homeostasis may provide a growth advantage to prostate cells in the absence of TRPM8, fostering a more tumorigenic environment. The downregulation of the steroid biosynthesis pathway, particularly genes like *Hsd17β7* and *Tm7sf2*, is notable, given the critical role of steroids in PC [29,30]. Hsd17β7 is essential for converting cholesterol precursors into steroids, including testosterone [29,30,31,32,33]. Its suppression aligns with previous findings from our study [15], which demonstrated elevated serum testosterone levels in TRPM8 KO mice. Impaired steroid biosynthesis may result in testosterone accumulation, enhancing AR-driven signaling, a well-established driver of PC progression [34,35].

This suggests that TRPM8 plays a key role in regulating androgen metabolism, and its loss may contribute to an androgen-rich environment that promotes tumor growth. The N-glycan biosynthesis pathway is significantly downregulated, with genes such as *Alg3*, *Alg8*, and *Dpm1* affected. N-glycosylation is critical for protein folding, stability, and function, especially for cell surface receptors involved in signal transduction. The dysregulation of glycosylation is a hallmark of cancer progression, facilitating cell detachment, migration, and immune evasion mechanisms that promote metastasis [36,37,38]. The small cell lung cancer pathway, with genes like *Akt2*, *Casp9*, and *E2f1* downregulated, further points to impaired apoptotic mechanisms and cell cycle control [39,40,41]. These genes are pivotal in regulating programmed cell death and ensuring proper cell division. Their suppression suggests a reduced ability of TRPM8 KO prostate cells to undergo apoptosis, potentially leading to unchecked proliferation and tumorigenesis. In summary, the downregulation of these pathways (Figure 2D and Table 2) underscores TRPM8’s extensive role in maintaining cellular homeostasis. The metabolic shifts and hormonal imbalances caused by TRPM8 loss, particularly the elevated testosterone levels and disrupted glycosylation, likely create a pro-tumorigenic environment that enhances cancer progression and metastasis in the prostate.

### 3.6. Functional Enrichment Analysis of Differentially Expressed Genes in TRPM8 Knockout Prostates Using Gene Ontology

**Figure 3:**
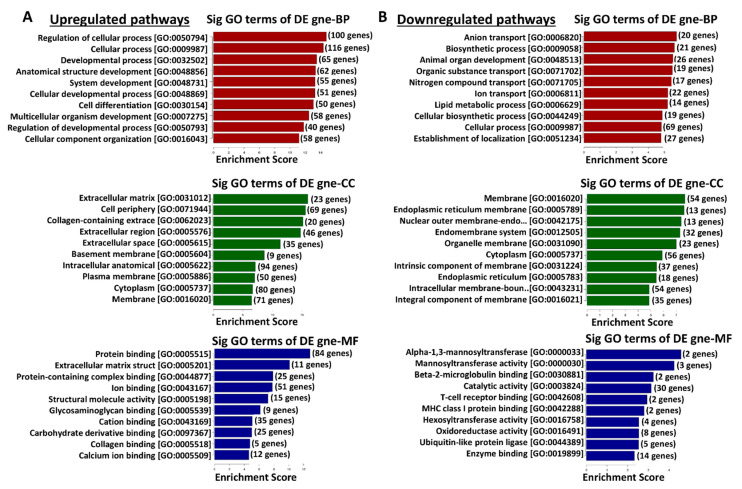
Gene ontology enrichment analysis of differentially expressed genes in TRPM8 KO mouse prostates. (**A**) The top panel displays the enriched GO terms for biological processes (BPs), cellular components (CCs), and molecular functions (MFs) for upregulated genes in TRPM8 KO compared to WT mouse prostates. Significant processes include the regulation of cell adhesion and ECM organization. (**B**) The bottom panel shows the enriched GO terms for BPs, CCs, and MFs for downregulated genes. Key processes involve metabolic pathways and structural components.

The gene ontology (GO) enrichment analysis for differentially expressed genes in TRPM8 KO prostates reveals distinct patterns of upregulated and downregulated pathways in various biological processes (BPs), cellular components (CCs), and molecular functions (MFs), as shown in Figure 3. In the TRPM8 KO prostates, upregulated pathways in BPs, including those related to cellular processes, developmental processes, and anatomical structure development, suggest enhanced cellular organization and differentiation activities. In CCs, increased expression in terms associated with the ECM, cell periphery, and plasma membrane indicates changes in cell–matrix interactions and cell surface organization. These upregulations are further supported by enrichment in MF terms related to protein binding, ECM structural constituents, and ion binding, suggesting adjustments in cell adhesion, signaling, and stability (Figure 3A). Conversely, downregulated pathways include BP terms associated with anion transport, biosynthetic processes, and lipid metabolic processes, indicating potential shifts in energy dynamics and a decrease in metabolic activity, which may alter cellular homeostasis. In CCs, the downregulation of terms related to the endoplasmic reticulum membrane, nuclear outer membrane, and cytoplasmic membrane suggests possible modifications in membrane structure and intracellular transport functions. Downregulated MF terms, such as mannosyltransferase and oxidoreductase activities, indicate changes in glycosylation and redox reactions, which may impact protein processing and receptor function in prostate cells (Figure 3B). These findings suggest that TRPM8 deletion in prostate tissue affects genes involved in structural organization and extracellular interactions while reducing activity in metabolic and biosynthetic processes. This dual regulation pattern may influence cellular function and tissue homeostasis in TRPM8 KO prostates.

### 3.7. Gene Set Enrichment Analysis (GSEA) of TRPM8 Knockout Prostates

**Figure 4:**
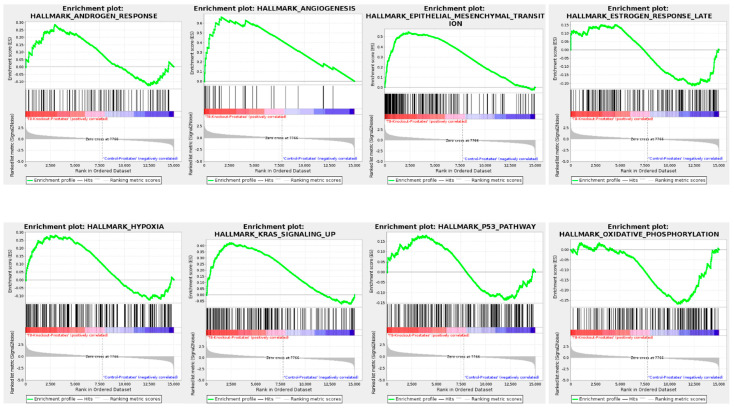
Hallmark pathways and gene ontology enrichment analysis in TRPM8 KO prostates. Displays the enrichment plots for hallmark pathways significantly altered in TRPM8 KO compared to WT mouse prostates. Key pathways include androgen response, angiogenesis, epithelial–mesenchymal transition, and oxidative phosphorylation.

**Figure 5:**
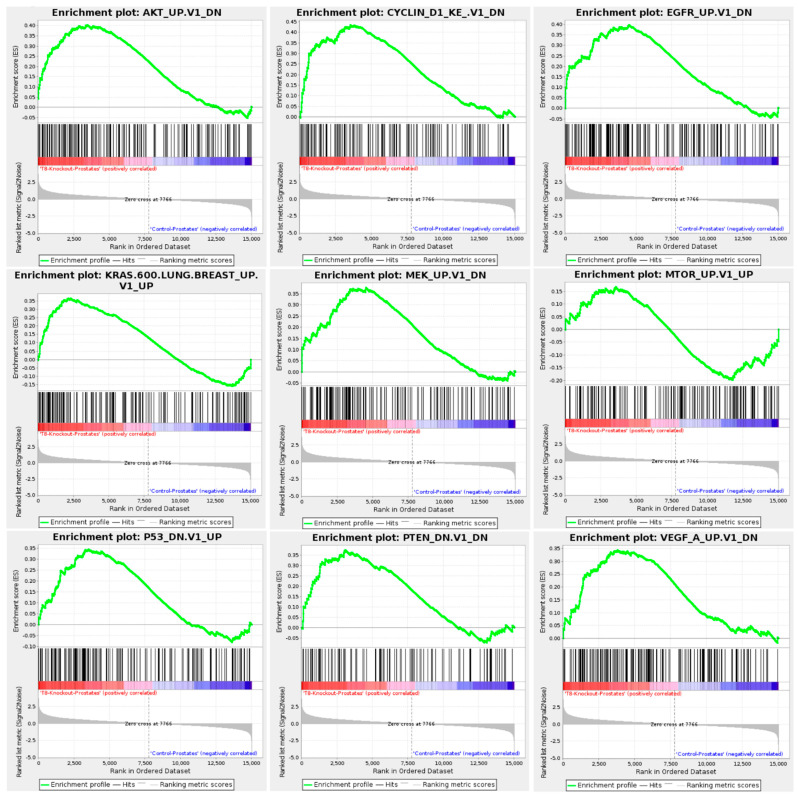
Gene Set Enrichment Analysis (GSEA) of specific TRPM8 KO mouse prostate signaling pathways. Displays enrichment plots for specific signaling pathways significantly altered in TRPM8 KO compared to WT mouse prostates. Key pathways include AKT, Cyclin D1, EGFR, KRAS, MEK, MTOR, P53, PTEN, and VEGF.

**Figure 6:**
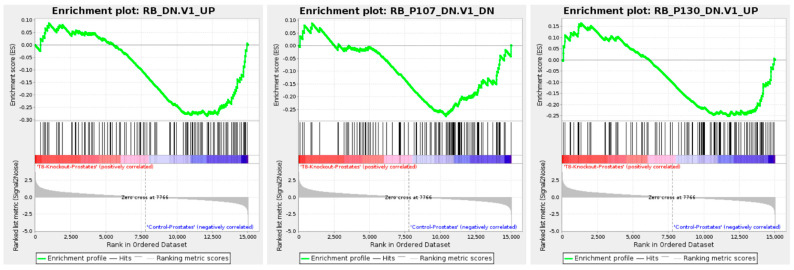
Retinoblastoma (RB) pathway enrichment analysis in TRPM8 KO mouse prostates. Displays the enrichment plot for the RB pathway, showing negative enrichment in TRPM8 KO compared to WT mouse prostates.

The GSEA for TRPM8 KO prostates highlights significant enrichment in hallmark pathways associated with cancer progression and cellular stress responses, as shown in Figure 4, Figure 5 and Figure 6.

In Figure 4, enriched hallmark pathways include androgen response, angiogenesis, epithelial–mesenchymal transition (EMT), estrogen response, and oxidative phosphorylation. The positive enrichment of androgen and estrogen response pathways in TRPM8 KO prostates aligns with elevated testosterone levels observed in TRPM8 KO mice, which may enhance AR-driven signaling [42]. The enrichment of EMT and angiogenesis pathways suggests a heightened potential for metastasis and blood vessel formation [43], both of which support a pro-tumorigenic environment in the absence of TRPM8. Additionally, pathways like hypoxia and oxidative phosphorylation indicate cellular adaptation to low oxygen conditions [44,45], a common feature in tumor tissues. Figure 5 highlights critical signaling pathways, including AKT, KRAS, MTOR, and MEK, which are essential for cell proliferation and survival. The positive enrichment of these pathways in TRPM8 KO prostates suggests increased proliferative and survival signaling, promoting oncogenic processes in the absence of TRPM8. Tumor suppressor pathways, such as PTEN and P53, also show positive enrichment, possibly indicating a compensatory mechanism [46,47]. The enrichment of PTEN and p53 pathways may represent a compensatory mechanism, where p53-mediated tumor suppression is activated in response to reduced PTEN activity [48].

Figure 6 examines the retinoblastoma (RB) pathways, specifically RB_DN_V1_UP, RB_P107_DN_V1_DN, and RB_P130_DN_V1_UP. These pathways show negative enrichment, indicating the downregulation of cell cycle regulatory mechanisms in TRPM8 KO prostates [49,50]. The reduced activity of RB-associated pathways, known for maintaining cell cycle control, may lead to increased cellular proliferation, aligning with the tumor-promoting signature seen across other pathways. Overall, the GSEA results suggest that TRPM8 deletion leads to the upregulation of pathways associated with cell proliferation, survival, metastasis, and angiogenesis. The concurrent activation of certain tumor suppressor pathways, such as PTEN and p53, highlights a possible compensatory response, particularly in light of the known relationship between PTEN loss and p53 upregulation in PC. This dual regulatory effect underscores TRPM8’s role in modulating key processes in PC progression. It suggests its loss may facilitate a more aggressive and tumorigenic environment while invoking cellular attempts to maintain growth control.

### 3.8. Differential Expression Profile and Correlation Analysis of TRPM8 Knockout Prostates

**Figure 7:**
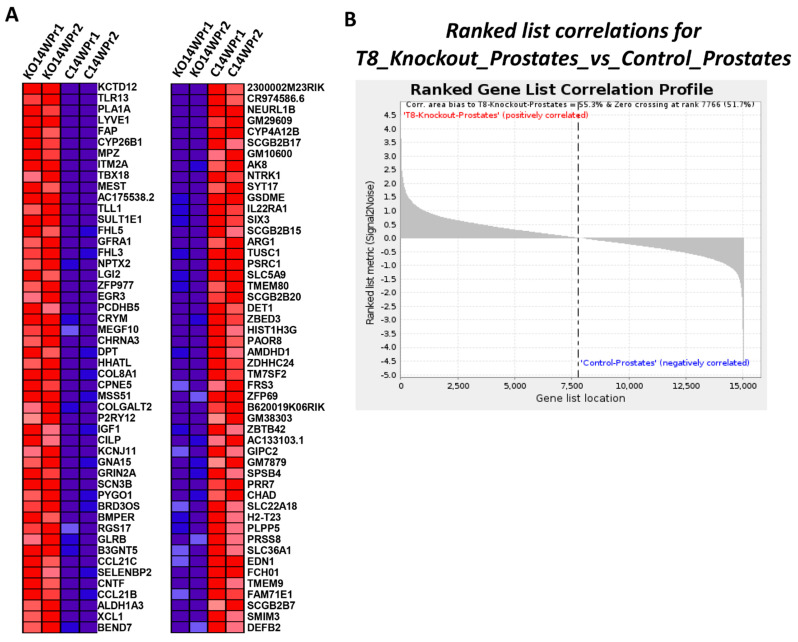
Ranked list correlations and heatmap analysis in TRPM8 KO vs. WT mouse prostates. (**A**) Displays the top 50 features for each phenotype in TRPM8 KO and WT mouse prostates. Red and blue colors indicate high and low expression levels, respectively. (**B**) Shows the ranked list correlations for TRPM8 KO prostates versus WT prostates. The plot illustrates the correlation bias toward TRPM8 KO prostates, with a positive correlation area of 55.3% and zero crossing at rank 7766 (51.7%).

In TRPM8 knockout (KO) prostates, a distinct expression profile is observed, with multiple genes showing significant upregulation or downregulation, as displayed in Figure 7A,B and Table 3. The heatmap (Figure 7A) illustrates the top 50 significantly expressed up- and downregulated genes, with upregulated genes in red and downregulated genes in blue, emphasizing the prominent changes in TRPM8 KO prostates compared to controls. Figure 7B presents the ranked gene list correlation profile, revealing a positive correlation bias toward TRPM8 KO prostates, with 55.3% of the gene list correlating positively and a zero-crossing point at rank 7766 (51.7%). This positive shift underscores the broad molecular impact of TRPM8 deletion on prostate gene expression.

Table 3 categorizes relevant genes by their molecular functions and association with cancer hallmarks, selected from all genes with a *p*-value threshold of ≤0.05 and a fold change cutoff of ±1.5. This table links gene expression changes to core processes implicated in cancer progression, including cell proliferation, apoptosis evasion, invasion and metastasis, angiogenesis, inflammation, ECM remodeling, and metabolic reprogramming. For instance, upregulated genes such as *TLR13*, *SERPINE1*, *MMP2*, and *FAP* are involved in immune signaling, inflammation, and ECM remodeling—key processes associated with tumorigenesis [50,51,52,53,54,55,56]. *TLR13*, a component of immune signaling networks, suggests an increased inflammatory response that could modify the tumor microenvironment to support cancer progression. Similarly, *SERPINE1* and *FAP* facilitate ECM remodeling, promoting cell migration and invasion. Additionally, the upregulation of VEGFD is indicative of enhanced angiogenesis, which supports tumor growth by facilitating new blood vessel formation.

Conversely, several downregulated genes, essential for maintaining tumor-suppressive functions, are identified. *CASP9* and *E2F1*, which are crucial for apoptosis and cell cycle control, respectively, exhibit reduced expression in TRPM8 KO prostates, potentially leading to decreased apoptotic activity and increased cellular proliferation, both hallmark traits of cancer cells. Downregulation in metabolic pathways is also observed, with genes like *CYB5R3* and *ALDH3B2* involved in redox balance and cellular metabolism, respectively [57,58], showing decreased expression. This implies disrupted metabolic homeostasis, which is essential for supporting the sustained growth of cancer cells. Together, Figure 7A,B and Table 3 illustrate how TRPM8 deletion leads to extensive shifts in gene expression, aligning with tumor-promoting mechanisms across various cancer-related pathways.

### 3.9. Coding Potential Analysis in TRPM8 Knockout Prostates

**Figure 8:**
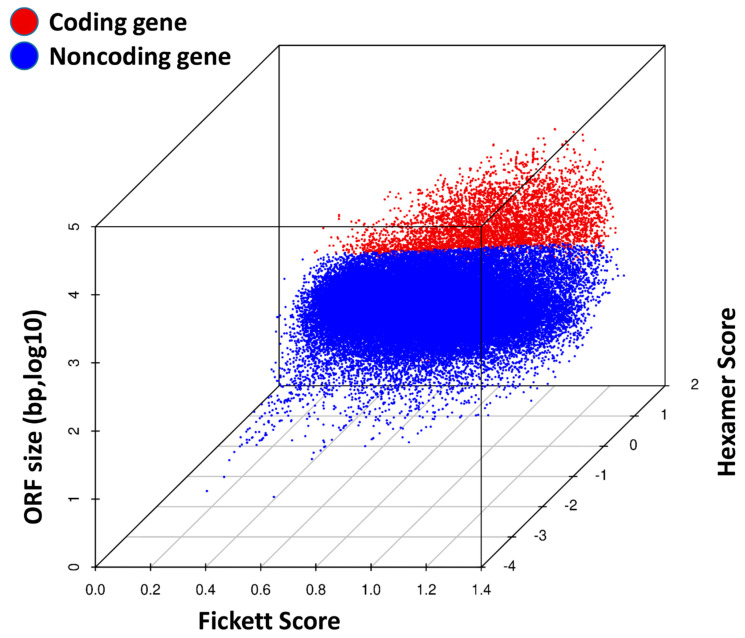
A 3D scatter plot of coding potential distribution in TRPM8 KO mouse prostates. The plot displays the distribution of coding and non-coding transcripts in TRPM8 KO mouse prostates. Red dots represent coding transcripts, while blue dots indicate non-coding transcripts. The axes show ORF size (bp, log10), Fickett score, and Hexamer score, which are used to assess coding potential.

The analysis of coding potential in TRPM8 KO mouse prostates demonstrates a clear distinction between coding and non-coding transcripts. In the 3D scatter plot (Figure 8), coding transcripts (red dots) cluster in areas with higher ORF sizes, Fickett scores, and Hexamer scores. In contrast, non-coding transcripts (blue dots) are generally associated with lower values in these metrics. This separation underscores the reliability of ORF size, Fickett scores, and Hexamer scores in effectively differentiating transcript types. The distinct distribution of coding and non-coding transcripts in TRPM8 KO prostates suggests that TRPM8 deletion may alter the expression profiles of both types, potentially impacting gene regulation and expression dynamics in the prostate tissue microenvironment. These coding and non-coding RNA expression changes could provide insights into the broader regulatory mechanisms influenced by TRPM8 loss, with implications for understanding altered gene function in PC progression.

### 3.10. Differential Expression of AR and TRPM8 in Castration-Sensitive and Castration-Resistant Prostate Cancer Models

**Figure 9:**
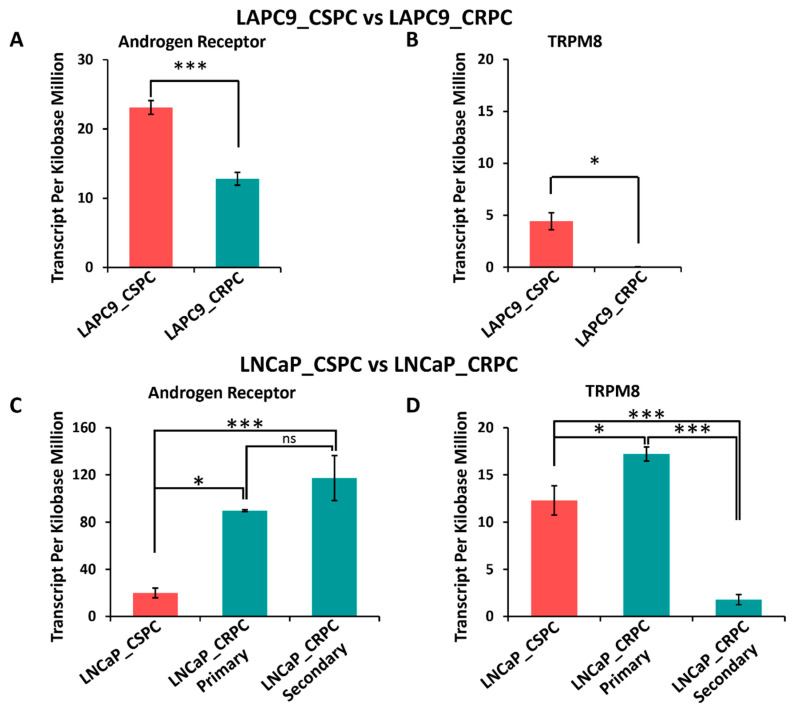
Gene expression in LAPC9 CSPC vs. CRPC and LNCaP CSPC vs. CRPC. The transcripts per kilobase million were obtained from the data extracted from the GSE88752 dataset and used to graph and compare the expression of the genes of interest: AR and TRPM8. (**A**) Expression of AR in LAPC9 CSPC is significantly higher than in CRPC (Student’s *t*-test: ***, *p* < 0.0001; *n* = 5). (**B**) Expression of TRPM8 in LAPC9 CSPC is significantly higher than in CRPC (Student’s *t*-test: *, *p* < 0.05; *n* = 5). (**C**) Expression of AR in LNCaP CSPC is significantly lower than in both primary and secondary CRPC (ANOVA with post hoc Tukey multiple comparisons of different cell types (ns, *p* > 0.05, *, *p* < 0.05, ***, *p* < 0.0001; *n* = 4). (**D**) Expression of TRPM8 LNCaP CSPC is significantly lower than in both primary and secondary CRPC (ANOVA with post hoc Tukey multiple comparisons of different cell types (*, *p* < 0.05, ***, *p* < 0.0001; *n* = 4).

Figure 9 illustrates the expression levels of the androgen receptor (AR) and TRPM8 in LAPC9 and LNCaP PC models under castration-sensitive (CSPC) and castration-resistant (CRPC) conditions. The gene expression profile was extracted with the help of PharmaFace (Begumpet, Hyderabad, India) using the GEO2R dataset (GSE88752). In the LAPC9 model, AR expression is significantly higher in CSPC compared to CRPC (Figure 9A, *p* < 0.0001), indicating a marked reduction in AR levels as tumors transition to a castration-resistant state (primary tumor). Similarly, TRPM8 expression is significantly reduced in CRPC compared to CSPC (Figure 9B, *p* < 0.05), suggesting that TRPM8 downregulation may be associated with the development of castration resistance in this model.

In contrast, the LNCaP model shows a different pattern of AR and TRPM8 expression. AR expression is significantly increased in CRPC primary tumors compared to CSPC (*p* < 0.05) and is further elevated in CRPC secondary tumors (enzalutamide resistant) (*p* < 0.0001; Figure 9C). However, there is no significant difference between primary and secondary CRPC (ns, *p* > 0.05; Figure 9C). TRPM8 expression in LNCaP cells shows an initial increase in CRPC primary tumors compared to CSPC (*p* < 0.05; Figure 9D) but is significantly reduced in CRPC secondary tumors compared to both CSPC and CRPC primary tumors (*p* < 0.0001; Figure 9D). This suggests that while TRPM8 may initially increase during early castration resistance, its subsequent loss could play a role in advanced disease progression, pointing to a different regulatory mechanism [6,59,60].

These distinct expression patterns suggest that the TRPM8-AR relationship may be influenced by other factors specific to each cell line, such as differences in AR isoform expression, co-factor availability, or epigenetic modifications. For instance, the reduction in TRPM8 in LAPC9 CRPC may reflect a direct role for TRPM8 in maintaining AR signaling under castration-sensitive conditions, while its loss facilitates resistance. Conversely, the dynamic changes in TRPM8 expression observed in LNCaP cells point to a more complex regulatory mechanism that may involve additional modulators of androgen signaling or tumor-specific adaptations. These context-dependent interactions underscore the complexity of TRPM8’s role in PC and highlight the need for further investigation into the specific mechanisms governing TRPM8-AR crosstalk in different cellular contexts.

## 4. Discussion

This study provides significant insights into the role of TRPM8 in PC progression, particularly its interaction with AR signaling and its impact on key oncogenic pathways. Our findings indicate that TRPM8 expression is markedly altered in PC tissues, showing a notable decline in metastatic samples compared to benign and early-stage tumors. This pattern suggests that TRPM8 may function as a tumor suppressor in the initial stages of PC, which is consistent with previous studies that have identified TRPM8 as a critical regulator of cell proliferation and apoptosis in PC cells [7,61].

The differential expression analysis revealed substantial changes in genes associated with critical cancer hallmarks, particularly those involved in ECM remodeling, such as *MMP2* and *FAP*. The upregulation of these genes in TRPM8 KO prostates suggests that the loss of TRPM8 enhances the metastatic potential of PC cells by promoting ECM degradation and facilitating cellular invasion. This aligns with findings from Grolez et al., which demonstrated that TRPM8 plays a role in inhibiting PC cell migration, indicating its potential as a therapeutic target [14].

Our pathway enrichment analysis further highlights significant alterations in key oncogenic pathways, including focal adhesion and PI3K-Akt signaling. The upregulation of these pathways in TRPM8 KO prostates indicates enhanced cell–matrix interactions and pro-survival signaling, which are critical for cancer progression and therapy resistance [11,62]. The concurrent downregulation of metabolic pathways and steroid biosynthesis suggests a metabolic reprogramming event, a hallmark of cancer that supports rapid proliferation and survival under stress conditions [63].

The Gene Set Enrichment Analysis (GSEA) revealed positive enrichment of androgen response, angiogenesis, and epithelial–mesenchymal transition (EMT) pathways in TRPM8 KO prostates. This finding is particularly relevant given our previous observation of elevated serum testosterone levels in TRPM8 KO mice. This suggests that TRPM8 loss may enhance AR-driven signaling and promote a more aggressive cancer phenotype [64,65]. The enrichment of angiogenesis and EMT pathways further supports the notion that TRPM8 deletion creates a pro-tumorigenic environment conducive to metastasis, corroborating earlier studies that have linked the reduction in TRPM8 activity to cancer cell migration and invasion [13,66].

We observed positive enrichment of oncogenic (AKT, KRAS, MTOR) and tumor suppressor (PTEN, P53) pathways in TRPM8 KO prostates. This seemingly paradoxical finding may reflect a complex compensatory mechanism, where the upregulation of PTEN and P53 pathways represents a cellular attempt to counteract the oncogenic signaling induced by TRPM8 loss. Previous studies have shown that PTEN inhibition can lead to p53 upregulation in PC as an adaptive response, highlighting the intricate balance between oncogenic and tumor suppressor pathways in cancer biology [47].

The AR and TRPM8 expression analysis of CSPC and CRPC models revealed a context–dependent relationship (Figure 9). The LAPC9 model showed parallel AR and TRPM8 reduction during the transition to CRPC. However, the LNCAP model displayed an inverse correlation, with TRPM8 downregulation in metastatic disease correlating with increased AR expression (Figure 1). This suggests a complex TRPM8-AR interaction in PC, with TRPM8 loss linked to advanced, castration-resistant settings [67,68]. This finding is consistent with previous studies implicating TRPM8 in androgen-dependent signaling, emphasizing the need to understand the regulatory mechanisms governing TRPM8 expression in different cancer contexts [69,70]. Based on these observations and our prior finding of AR-mediated TRPM8 degradation [6,7,15], we propose the following model. (1) In early-stage, hormone-sensitive PC (e.g., LNCaP cells), AR can induce TRPM8 mRNA expression. However, the TRPM8 protein is unstable and targeted for ubiquitination-mediated degradation, even in the presence of AR. This is supported by our previous findings that AR inhibition with hydroxyflutamide rescues TRPM8 from degradation and restores its plasma membrane localization. (2) In advanced, metastatic, and castration-resistant settings (e.g., PC3 cells), TRPM8 expression is often lost. This may be due to a number of factors, including altered AR expression, changes in chromatin accessibility at the TRPM8 locus, or the involvement of alternative regulatory mechanisms. The absence of AR in PC3 cells is correlated with no TRPM8 mRNA expression, which suggests AR dependence for mRNA expression. In addition, the overexpression of the TRPM8 protein in PC3 cells can induce calcium-mediated cytotoxicity and apoptosis. (3) This TRPM8 loss can enhance AR activity, providing a mechanism for castration resistance. (4) TRPM8-AR interactions vary based on cellular context. The downregulation of retinoblastoma (RB) pathway genes in TRPM8 KO prostates suggests a potential mechanism for enhanced cell cycle progression and reduced growth control. Inactivation of the RB pathway is a common feature in advanced PC and is associated with poor prognosis [71,72]. Our findings indicate that TRPM8 loss may contribute to this process, further promoting cancer progression.

Moreover, the coding potential analysis revealed distinct clustering of coding and non-coding transcripts in TRPM8 KO prostates, suggesting that TRPM8 deletion not only affects protein-coding genes but also impacts the expression of non-coding RNAs, which are increasingly recognized as important regulators of cancer-related processes [73,74,75,76,77]. Future studies should investigate the specific roles of these differentially expressed non-coding RNAs in PC progression.

Our comprehensive analysis of TRPM8 KO prostates reveals a multifaceted role for TRPM8 in PC biology. The loss of TRPM8 appears to create a pro-tumorigenic environment characterized by enhanced AR signaling, increased ECM remodeling, and alterations in key oncogenic and tumor suppressor pathways. These findings suggest that TRPM8 may function as a molecular brake on PC progression, and its loss could contribute to the development of more aggressive disease phenotypes. The complex interplay between TRPM8 and AR signaling, as well as the context-dependent effects observed in different cell models, underscores the need for further research to fully elucidate the mechanisms by which TRPM8 influences PC progression. Future studies should focus on validating these findings in human PC samples and exploring the potential of TRPM8 as a therapeutic target or biomarker for PC progression and treatment response.

## Figures and Tables

**Table 1:** KEGG pathway enrichment analysis of upregulated genes in TRPM8 knockout mouse prostates. It lists the top enriched KEGG pathways for upregulated genes in TRPM8 KO compared to WT mouse prostates. It includes pathway ID, definition, Fisher *p*-value, and associated gene symbols.

Pathway ID	Definition	Fisher *p*-Value	Genes
mmu04510	Focal_adhesion	0.00283935	*Col4a5//Flnc//Igf1//Lama2//Lamb1//Pdgfra*
mmu05410	Hypertrophic_cardiomyopathy	0.003817752	*Igf1//Lama2//Myl3//Tpm2*
mmu05414	Dilated_cardiomyopathy	0.004288076	*Igf1//Lama2//Myl3//Tpm2*
mmu05146	Amoebiasis	0.00677927	*Col4a5//Gna15//Lama2//Lamb1*
mmu05145	Toxoplasmosis	0.00746598	*Hspa1a//Hspa1b//Lama2//Lamb1*
mmu04213	Longevity_regulating_pathway-multiple_species	0.009447223	*Hspa1a//Hspa1b//Igf1*
mmu04151	PI3K-Akt_signaling_pathway	0.012866392	*Angpt1//Col4a5//Igf1//Lama2//Lamb1//Lpar1//Pdgfra*
mmu04010	MAPK_signaling_pathway	0.017163356	*Angpt1//Flnc//Hspa1a//Hspa1b//Igf1//Pdgfra*
mmu04015	Rap1_signaling_pathway	0.017293189	*Angpt1//Igf1//Lpar1//Pdgfra//Rapgef4*
mmu04261	Adrenergic_signaling_in_cardiomyocytes	0.022211878	*Myl3//Rapgef4//Scn7a//Tpm2*
mmu04512	ECM-receptor_interaction	0.024093049	*Col4a5//Lama2//Lamb1*
mmu04610	Complement_and_coagulation_cascades	0.027795597	*C7//Cfh//Pros1*
mmu05222	Small_cell_lung_cancer	0.027795597	*Col4a5//Lama2//Lamb1*
mmu04061	Viral_protein_interaction_with_cytokine_and_cytokine_receptor	0.029357007	*Ccl21a//Ccl21b//Ppbp*
mmu05200	Pathways_in_cancer	0.036273337	*Apc//Col4a5//Igf1//Lama2//Lamb1//Lpar1//Pdgfra//Pmaip1*
mmu04064	NF-kappa_B_signaling_pathway	0.03784812	*Bcl2a1d//Ccl21a//Ccl21b*
mmu04974	Protein_digestion_and_absorption	0.040615603	*Col14a1//Col4a5//Col8a1*
mmu04066	HIF-1_signaling_pathway	0.046450354	*Angpt1//Eno1b//Igf1*

**Table 2:** Downregulated pathway genes in TRPM8 KO mice compared to WT mouse prostates. This table lists the significantly downregulated pathways in TRPM8 KO mouse prostates compared to WT controls. Each entry includes the KEGG pathway ID, definition, Fisher *p*-value, and associated gene symbols.

Pathway ID	Definition	Fisher *p*-Value	Genes
mmu05222	Small_cell_lung_cancer	0.001001771	*Akt2//Casp9//Cks2//E2f1*
mmu00510	N-Glycan_biosynthesis	0.001747349	*Alg3//Alg8//Dpm1*
mmu00100	Steroid_biosynthesis	0.004082726	*Hsd17b7//Tm7sf2*
mmu05167	Kaposi_sarcoma-associated_herpesvirus_infection	0.004227729	*Akt2//Casp9//E2f1//H2-K1//H2-T23*
mmu05169	Epstein-Barr_virus_infection	0.004729153	*Akt2//Casp9//E2f1//H2-K1//H2-T23*
mmu05223	Non-small_cell_lung_cancer	0.004939961	*Akt2//Casp9//E2f1*
mmu01100	Metabolic_pathways	0.004987554	*Acyp1//Aldh3b2//Alg3//Alg8//Amdhd1//Arg1//Cbs//Cmpk2//Cyp4a12b//Dpm1//Hsd17b7//Lipt1//Pigp//Sqor//Tm7sf2*
mmu05212	Pancreatic_cancer	0.005745739	*Akt2//Casp9//E2f1*
mmu00340	Histidine_metabolism	0.006856938	*Aldh3b2//Amdhd1*
mmu05163	Human_cytomegalovirus_infection	0.007282135	*Akt2//Casp9//E2f1//H2-K1//H2-T23*
mmu05416	Viral_myocarditis	0.008351367	*Casp9//H2-K1//H2-T23*
mmu04218	Cellular_senescence	0.011345937	*Akt2//E2f1//H2-K1//H2-T23*

**Table 3:** Key upregulated and downregulated genes in TRPM8 KO prostates organized by cancer hallmarks. This table provides a selection of significantly upregulated and downregulated (a 1.5-fold change cutoff and a *p*-value > 0.05) genes in TRPM8 KO prostates categorized by their molecular functions and roles in cancer hallmarks.

Gene	Direction	Molecular Function	Implication in Cancer
*TLR13*	Upregulated	Immune signaling	Enhances inflammatory response, tumor microenvironment modification
*CXCL1*	Upregulated	Chemokine activity	Promotes inflammation and attracts immune cells, supports metastasis
*SERPINE1*	Upregulated	Extracellular matrix remodeling	Involved in invasion and metastasis through ECM interactions
*FAP*	Upregulated	ECM remodeling	Supports tumor invasion and cell migration
*VEGFD*	Upregulated	Angiogenesis	Promotes blood vessel formation, facilitating tumor growth
*COL8A1*	Upregulated	Structural constituent of ECM	Aids in ECM structure, supporting metastasis
*CYB5R3*	Downregulated	Redox balance	Impaired cellular metabolism and oxidative stress response
*CASP9*	Downregulated	Apoptosis initiation	Reduced apoptosis, leading to unchecked cell survival
*E2F1*	Downregulated	Cell cycle regulation	Impaired cell cycle control, promoting proliferation
*Aldh3b2*	Downregulated	Metabolism	Altered cellular energy metabolism
*Hsd17β7*	Downregulated	Steroid biosynthesis	Alters hormonal balance, may support tumorigenesis

## Data Availability

The data will be made publicly available upon publication of this manuscript. The large data sequencing files will be shared upon valid request.

## Supplementary Materials

The following supporting information can be downloaded at https://www.mdpi.com/article/10.3390/cells14070501/s1. Figure S1: RNA quality and sequencing workflow for TRPM8 knockout and wild-type mouse prostates.
