# Supplementary material for: The Impact of TRPM8 on Prostate Cancer Transcriptomic Dynamics
Source: Cells. 2025 Mar 27;14(7):501. doi: 10.3390/cells14070501 (PMC11988096; doi:10.3390/cells14070501)
Supplement: Supplementary file 1 [file cells-14-00501-s001.zip › cells-3437105-supplementary.pdf]

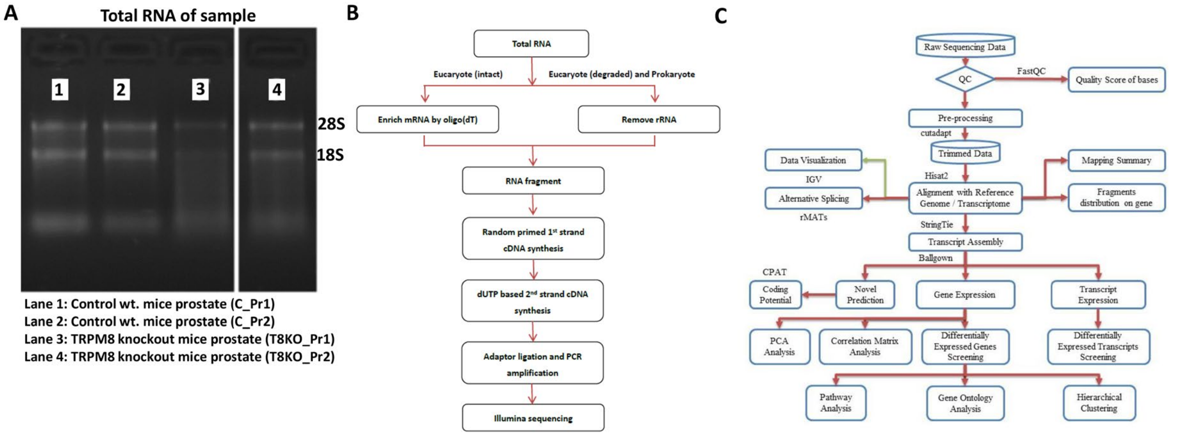

Scheme S1. RNA quality and sequencing workflow for TRPM8 knockout and wild-type mouse prostates. A. Agarose gel electrophoresis showing RNA integrity for control wild-type (WT) and TRPM8 KO mouse prostates. Lanes 1 and 2 represent WT samples, while lanes 3 and 4 represent TRPM8 KO samples, indicating high RNA quality with distinct 28S and 18S rRNA bands. **B.** Schematic of the RNA-seq workflow process, including RNA extraction, library preparation, sequencing, and data analysis steps to assess differential gene expression between WT and TRPM8 KO prostate tissues. **C.** Flowchart detailing bioinformatic analysis steps, including quality control, mapping to the reference genome, differential expression analysis, and pathway enrichment to identify TRPM8's role in PC.
